# Using Long Short-Term Memory and Causal Forest to Identify Preeclampsia Subtypes with Differential Aspirin Associations for Preterm Birth Prevention

**DOI:** 10.3390/bioengineering13101171

**Published:** 2026-10-08

**Authors:** Cheng Liu, Yang Su, Yao Wei, Yongxin Li, Chengxi Bao, Rui Qiao

**Affiliations:** Department of Clinical Laboratory, Peking University Third Hospital, Beijing 100191, China; chengliu0720@outlook.com (C.L.); baochengxi2026@163.com (C.B.)

**Keywords:** preeclampsia, aspirin, preterm birth, long short-term memory, causal forest, liver and coagulation abnormalities

## Abstract

Aspirin prevents preterm birth in some women with preeclampsia but not others, yet no longitudinal multi-indicator study has examined subtype differences in aspirin response. We aimed to identify subtypes most likely to benefit. In this retrospective cohort of 53,362 deliveries (4505 preeclamptic women), we applied a two-stage machine learning framework using 15 indicators (14 laboratory tests plus systolic blood pressure) from two-time windows: before 16 weeks and within 2 weeks before delivery. A long short-term memory autoencoder with K-means clustering identified subtypes, and causal forest estimated the average treatment effect of aspirin on preterm birth (<37 weeks) for each subtype, adjusted for confounders. Five stable subtypes were identified (silhouette coefficient 0.542; mean adjusted Rand index 0.903). One subtype (*n* = 345), characterised by mild liver enzyme elevation and coagulation abnormalities in late pregnancy, had the highest preterm birth rate (57.7%) and ICU admission rate (9.6%), and showed the largest aspirin-associated reduction in preterm birth (ATE −0.026, 95% CI: −0.032 to −0.021), consistent across 70/30 splits. Sensitivity analyses using 34 indicators confirmed similar effects (ATE −0.027, 95% CI: −0.031 to −0.023), while no benefit was observed in non-preeclamptic women. These findings suggest that aspirin prophylaxis may be associated with a greater reduction in preterm birth in this preeclampsia subtype, but further validation is required.

## 1. Introduction

Preeclampsia (PE) is one of the leading causes of maternal and neonatal mortality and is closely associated with adverse cardiovascular and neurological development in newborns after birth [1]. The diagnostic criteria are new-onset hypertension after 20 weeks of gestation accompanied by any evidence of organ dysfunction, including renal (proteinuria, elevated creatinine), hepatic (elevated liver enzymes), coagulation (thrombocytopenia), neurological (headache, visual disturbances, etc.), or pulmonary edema [2]. Clinically, PE may involve single or multiple organs, and some patients present with atypical manifestations such as edema, abdominal pain, or skin rash [3]. This variability underscores that PE is not a uniform disease but a spectrum of phenotypes with distinct underlying pathophysiological processes.

Currently, the classification of PE is mainly based on the timing of onset. PE can be divided into early-onset (occurring before 34 weeks) and late-onset (occurring after 34 weeks). According to the timing of delivery, it can be classified as preterm-onset (before 37 weeks), term-onset (after 37 weeks), and postpartum-onset [1]. However, these temporal classifications do not capture the heterogeneity in organ injury patterns, which may have greater pathophysiological and prognostic relevance. Previous studies have attempted to classify PE into subtypes such as renal impairment, liver impairment, coagulation disorders, and cardiac injury using antenatal laboratory results [4]. Longitudinal trajectories of single or two indicators in PE have also been previously studied [5,6,7]. Nevertheless, studies using multiple indicators based on longitudinal data for subtyping are still lacking. Machine learning methods [8,9], such as long short-term memory (LSTM), have advantages in modeling the longitudinal changes in multiple indicators [10,11,12,13].

Multiple studies have confirmed that aspirin use before 16 weeks of gestation can prevent PE [14,15]. Its use reduces the incidence of preterm birth by approximately 50% in high-risk pregnant women. However, for low-risk women, the preventive effect of aspirin is poor, with a number needed to treat (NNT) as high as several thousand [16]. This heterogeneity in treatment response highlights the urgent need to identify which PE patients are most likely to benefit from aspirin prophylaxis, thereby enabling more personalised and cost-effective prevention strategies.

Based on the above background, we used longitudinal pregnancy data to classify PE patients into subtypes according to their distinct patterns of organ injury and to evaluate the preventive effect of aspirin across these subtypes.

## 2. Methods

### 2.1. Study Design

Using longitudinal pregnancy laboratory indicators, PE subtypes were identified through LSTM training, and causal forest [17] was used to evaluate the preventive effect of aspirin on preterm birth across different subtypes.

### 2.2. Study Population

The study included 53,362 pregnant women who delivered at our hospital between 1 January 2017 and 31 August 2025. Based on discharge diagnosis using ICD-10 codes (O11, O14), 4505 women with PE and 48,857 non-PE pregnant women were identified. The diagnostic criteria for PE at our hospital were consistent with the ACOG guidelines [2], including new-onset hypertension (systolic blood pressure ≥ 140 mmHg and/or diastolic blood pressure ≥ 90 mmHg) after 20 weeks of gestation, accompanied by any of the following: urinary protein quantification ≥ 0.3 g/24 h, urinary protein-to-creatinine ratio ≥ 0.3, or random urine protein ≥(+); or in the absence of proteinuria, accompanied by any of the following evidence of organ or system involvement: cardiac, pulmonary, hepatic, renal, hematological, digestive, or neurological abnormalities, or placental-fetal compromise. All included women had antepartum PE.

### 2.3. Laboratory Indicators

All laboratory results were obtained from routine tests at our hospital, with daily quality control data within acceptable ranges. Blood pressure measurements and aspirin use records from outpatient follow-up medical records were used for subsequent analysis. To reduce bias due to excessive missing data, two fixed time windows were defined: before 16 gestational weeks and within 2 weeks prior to delivery. Repeated indicators within each window were averaged. Details of missing data are provided in Supplemental Table S1. A total of 14 laboratory indicators plus blood pressure were included in the primary analysis, and 32 laboratory indicators (with <50% missing values) plus 24-h urinary protein quantification and blood pressure were included in the sensitivity analysis.

### 2.4. LSTM Training and K-Means Clustering

Data were organized for each patient with 15 indicators at two time points. Missing values were filled with −999 as a placeholder to maintain the fixed two-time-point tensor structure required by the LSTM autoencoder [18]. A binary mask was used to distinguish observed from missing entries; −999 served only as a structural placeholder, and masked mean squared error (MSE) ensured that missing positions did not directly contribute to the reconstruction loss. Because −999 could still influence the learned representation, this approach was considered pragmatic and conservative rather than fully unbiased. To evaluate this missing data strategy, multiple imputation followed by repeated LSTM training and K-means clustering was performed as a sensitivity analysis.

An LSTM autoencoder was constructed: the encoder used a single-layer LSTM (15 units) to compress the two-time steps into a 15-dimensional embedding vector. The loss function was masked MSE, which computed reconstruction error only for non-masked positions. The model was optimized using Adam with a batch size of 15, trained for 100 epochs, and early stopping was applied (training stopped if validation loss did not improve for 10 consecutive epochs). After training, the 15-dimensional embedding vectors output by the encoder were extracted, and K-means clustering was performed to identify patient subtypes. The optimal number of clusters was determined within a range of 2 to 6 using the silhouette coefficient. Each patient was assigned a cluster label, and the results were visualized using principal component analysis (PCA) for dimensionality reduction.

### 2.5. Causal Forest

Clusters were learned from the full dataset, and the 70/30 split was used mainly for causal forest validation. Causal forest was used to estimate the individual treatment effect of aspirin on preterm birth [19,20,21]. The causal estimand was the association between aspirin exposure and preterm birth among women who already developed preeclampsia, rather than the preventive effect of aspirin on preeclampsia incidence in the broader at-risk pregnant population. Covariates included demographic characteristics (age, parity, gravidity, pre-pregnancy body mass index (BMI), diabetes, hypertension) and 15 indicators measured before 16 gestational weeks. These covariates were selected a priori based on clinical relevance and previous retrospective studies. No formal validation of causal assumptions, such as positivity or covariate balance diagnostics, was performed because of the retrospective nature of the study. As a retrospective study, this design cannot control all sources of bias. Because aspirin use was not randomized, the assumption of no unmeasured confounding cannot be fully verified, and unmeasured factors such as aspirin dose, timing of initiation, adherence, and prescribing indication may cause residual confounding. Missing values for early indicators were imputed with the median. Individual treatment effects were estimated, and the average treatment effect (ATE) was calculated for each cluster, with covariate importance ranking provided. Subsequently, the data were randomly split into training (70%) and validation (30%) sets. The causal forest model was trained on the training set and then applied to the validation set to calculate the ATE for each cluster in both sets.

To assess covariate balance and overlap within each cluster, we computed standardized mean differences (SMD) for baseline covariates between aspirin-exposed and unexposed women. We also estimated propensity scores [22,23] using logistic regression with aspirin use as the outcome and age, BMI, gravidity, parity, diabetes, hypertension, and preeclampsia history as covariates [24]. Propensity score distributions and common support regions were examined for each cluster.

### 2.6. Sensitivity Analyses

For the initially obtained 15-indicator LSTM results, bootstrap resampling with replacement was performed 100 times. After each resampling, clustering was repeated, and the Hungarian algorithm was used to match the original clusters with the resampled clusters. The average Jaccard similarity for each cluster was calculated to assess clustering stability. Subsequently, 10 bootstrap resampling iterations (each with replacement at the same sample size as the full dataset) were conducted to repeatedly train the LSTM autoencoder, with the number of clusters fixed at 5. The adjusted Rand index (ARI) between the resulting cluster labels and the initial clustering results was calculated to evaluate the stability of LSTM training. Using the 34 indicators with a missing rate below 50%, the LSTM autoencoder was reconstructed: the encoder used a single-layer LSTM (34 units), the batch size was set to 34 during training, and all other parameters remained the same as before. Finally, LSTM training was performed in the non-PE population using the same 15 indicators and parameters as those used in the PE population. After clustering, the ATE for each cluster was calculated using causal forest. To evaluate the impact of missing values on training results, data from the second multiple imputations were used for LSTM training and K-means clustering, and the ARI was calculated after repeating the LSTM training 10 times following the above steps. These sensitivity analyses support robustness but do not resolve causal or clinical-validity concerns.

### 2.7. Statistical Analysis

For group comparisons, one-way analysis of variance (ANOVA) was used for continuous variables that followed a normal distribution; the Kruskal–Wallis test was used for continuous variables that did not follow a normal distribution or had a sample size greater than 5000. Categorical variables were compared using the chi-square test. All statistical analyses were performed in R version 4.3.3 (R Core Team, 2024), primarily using the base stats package. Data cleaning and causal forest modeling were conducted using the following R packages: dplyr, grf, ggplot2, caret, lubridate, readxl, and tidyr. LSTM autoencoder training and K-means clustering were performed in PyCharm 2024.1.1 using the following Python packages (PyCharm 2024.1.1): tensorflow, numpy, pandas, matplotlib, and scikit-learn.

## 3. Results

### 3.1. Baseline Characteristics

**Figure 1:**
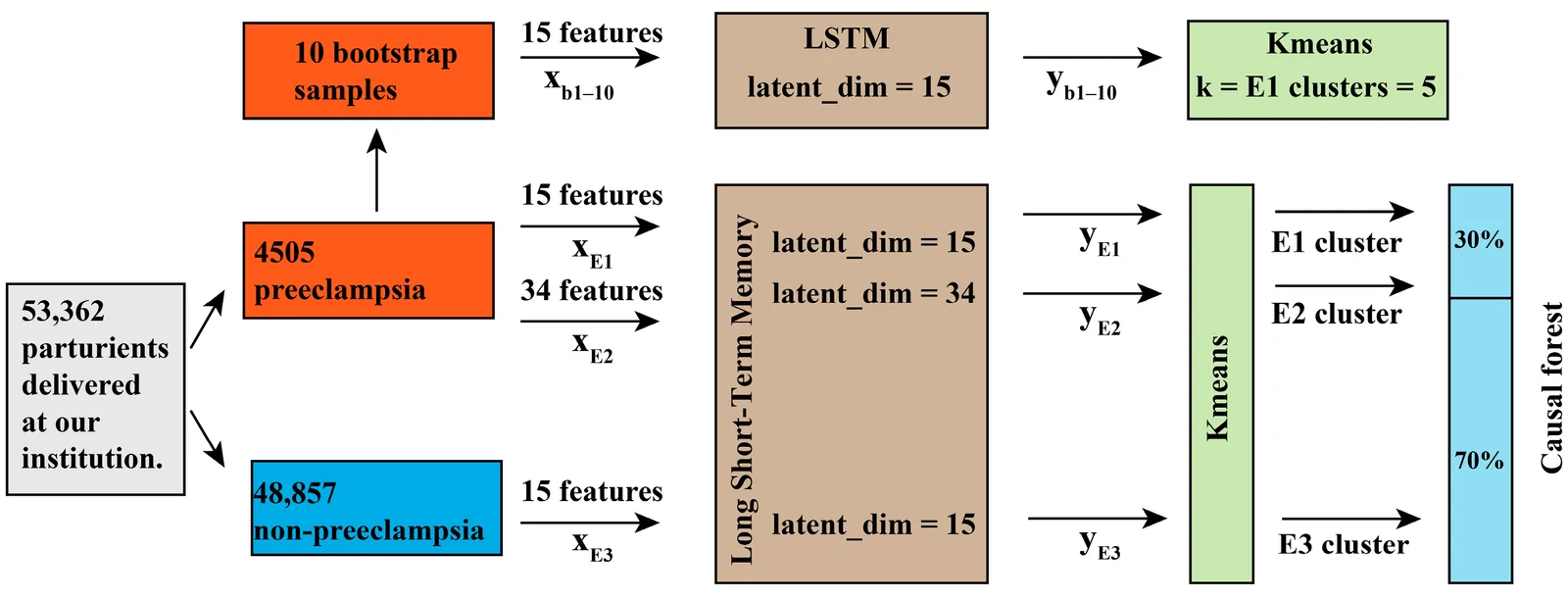
Study workflow. From 53,362 deliveries, 4505 PE patients and 48,857 non-PE women were included. Two feature sets (15 and 34 indicators) were used. LSTM autoencoders (latent dimensions 15 or 34) were trained, followed by K-means clustering (k = 5, determined by silhouette coefficient). Cluster stability was evaluated by 10 bootstrap resampling with LSTM retraining and ARI. Causal forest (70/30 train/validation split after clustering) assessed aspirin’s effect on preterm birth for each cluster. Non-PE controls were analyzed using the same 15-feature model.

The experimental design workflow is shown in Figure 1. PE patients were older (33, 31–36 vs. 33, 30–36 years, *p* < 0.001), had greater postpartum blood loss (730, 570–1060 vs. 650, 550–890 mL, *p* < 0.001), had higher pre-pregnancy BMI (23.9, 21.5–26.8 vs. 21.7, 20.0–23.9 kg/m^2^, *p* < 0.001), and had shorter gestational age at delivery (260, 244–270 vs. 274, 267–280 days, *p* < 0.001) compared with non-PE women. However, no differences were observed between the two groups in postpartum platelet, plasma, or red blood cell transfusion. The proportions of diabetes (36.2% vs. 27.4%, *p* < 0.001), hypertension (25.7% vs. 8%, *p* < 0.001), and placental abruption (3% vs. 0.6%, *p* < 0.001) were significantly higher in the PE group than in the control group. In addition, the PE group had a higher rate of intensive care unit (ICU) admission (3.1% vs. 0.6%, *p* < 0.001) and a higher rate of aspirin use (16.2% vs. 9.6%, *p* < 0.001). The proportions of multiple pregnancy (25.2% vs. 8.3%, *p* < 0.001) and neonatal death (2.4% vs. 1.4%, *p* < 0.001) were also higher in the PE group. Results are shown in Table 1.

### 3.2. LSTM Stably Classified Pregnant Women Diagnosed with PE into Five Subtypes

**Figure 2:**
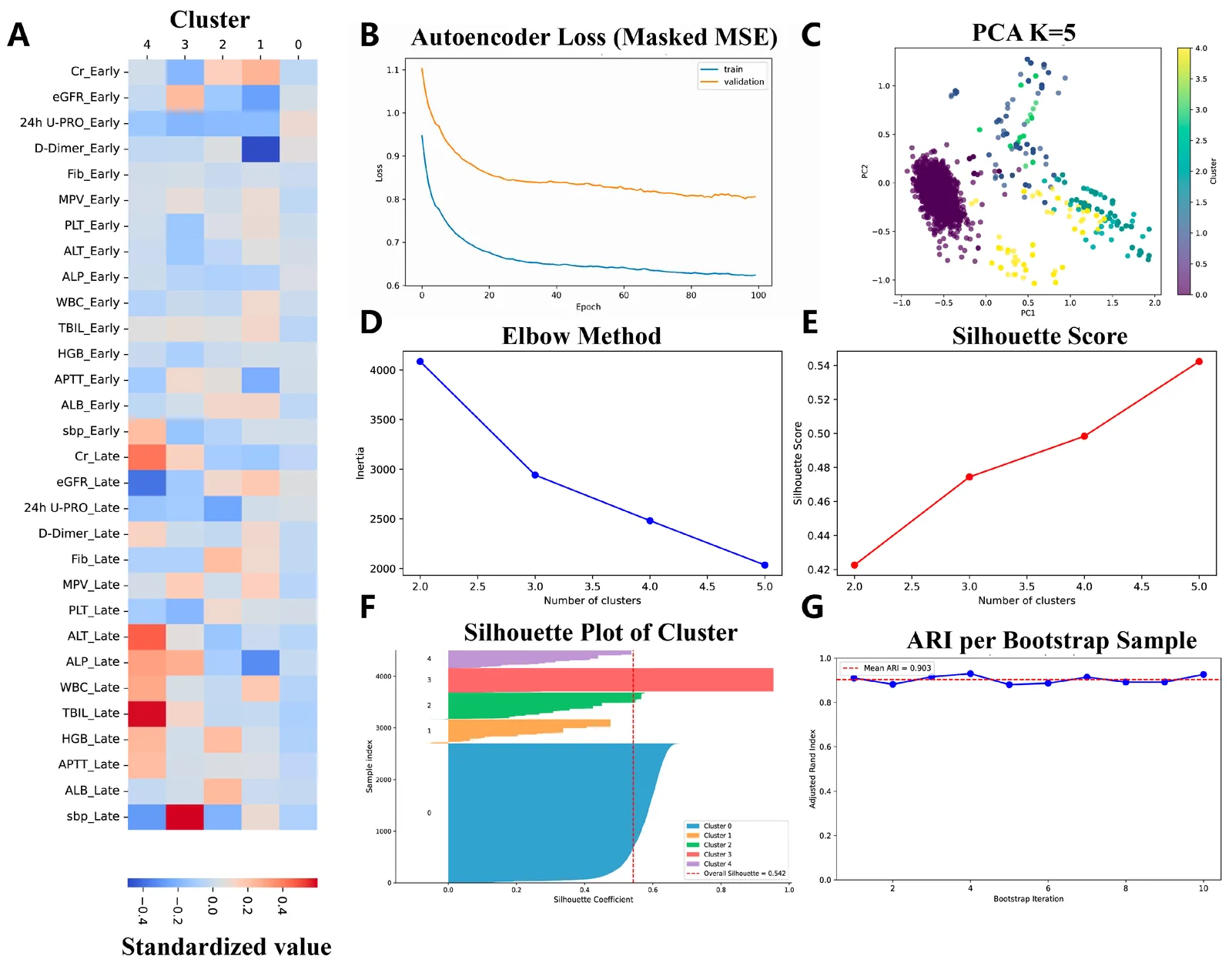
LSTM autoencoder training and K-means clustering results using 15 indicators from two-time windows. (**A**) heatmap showing standardized mean values (z-scores) of the 15 indicators across the five identified clusters; rows represent indicators and columns represent clusters. (**B**) training and MSE curves during LSTM autoencoder training, indicating model convergence without overfitting; early stopping was applied. (**C**) PCA visualization of the 15-dimensional LSTM embedding space, with points colored by cluster assignment. (**D**) Elbow method showing inertia for k = 2–6. (**E**) Silhouette coefficient for k = 2–6; although k = 6 had a slightly higher silhouette coefficient (0.55) than k = 5 (0.542), k = 6 produced smaller and less stable clusters, and k = 5 was therefore selected. (**F**) Silhouette plot for each cluster at k = 5. (**G**) ARI calculated from 10 repeated LSTM trainings with bootstrap resampling, indicating clustering stability. Cluster sizes were *n* = 2696 (Cluster 0), *n* = 471 (Cluster 1), *n* = 516 (Cluster 2), *n* = 476 (Cluster 3), and *n* = 345 (Cluster 4).

Using 15 indicators at two-time windows (before 16 gestational weeks and within 2 weeks prior to delivery), LSTM training followed by K-means clustering yielded five subtypes. The PCA plot is shown in Figure 2C. The silhouette coefficient was 0.542 (Figure 2D–F). Cluster 4 was characterized by an increase in alanine aminotransferase (ALT), alkaline phosphatase (ALP), total bilirubin (TBIL), and D-dimer from early pregnancy (before 16 gestational weeks) to the prenatal period (within 2 weeks before delivery), along with a decrease in platelets and fibrinogen (Figure 2A; Supplemental Figure S1). After 100 bootstrap resampling iterations, the overall Jaccard similarity for each cluster was 0.998. Subsequently, 10 bootstrap resampling iterations were performed to retrain the LSTM model with the number of clusters fixed at 5. The ARI between each resampled clustering result and the initial clustering result was calculated (Figure 2G), with a mean ARI of 0.903.

Using 32 indicators with <50% missing values, plus 24-h urinary protein quantification and blood pressure, LSTM training and K-means clustering were repeated, yielding 5 clusters with a silhouette coefficient of 0.29. A cluster with similar features (liver enzyme elevation and coagulation abnormalities) again emerged as Cluster 4. Subsequently, 10 bootstrap resampling iterations were performed to retrain the LSTM model; the mean ARI among the clusterings obtained from each iteration was 0.802 (Supplemental Figure S3). In summary, using laboratory indicators from two-time windows, LSTM training stably identified a subgroup of PE patients characterized by liver enzyme elevation and coagulation abnormalities.

To account for the effect of different missing data handling methods on LSTM training and clustering results, we applied multiple imputation and used the second imputed dataset for repeated LSTM training. The results are shown in Supplemental Figure S6. Although a cluster characterized by liver enzyme elevation and coagulation abnormalities was still observed after imputation, the stability of LSTM training and clustering decreased substantially (mean ARI = 0.18, SD = 0.34), suggesting that multiple imputation may have altered the characteristics of the data.

### 3.3. Aspirin Was Associated with the Largest Estimated Reduction in Preterm Birth in PE Patients Characterized by Liver Enzyme Elevation and Coagulation Abnormalities

**Figure 3:**
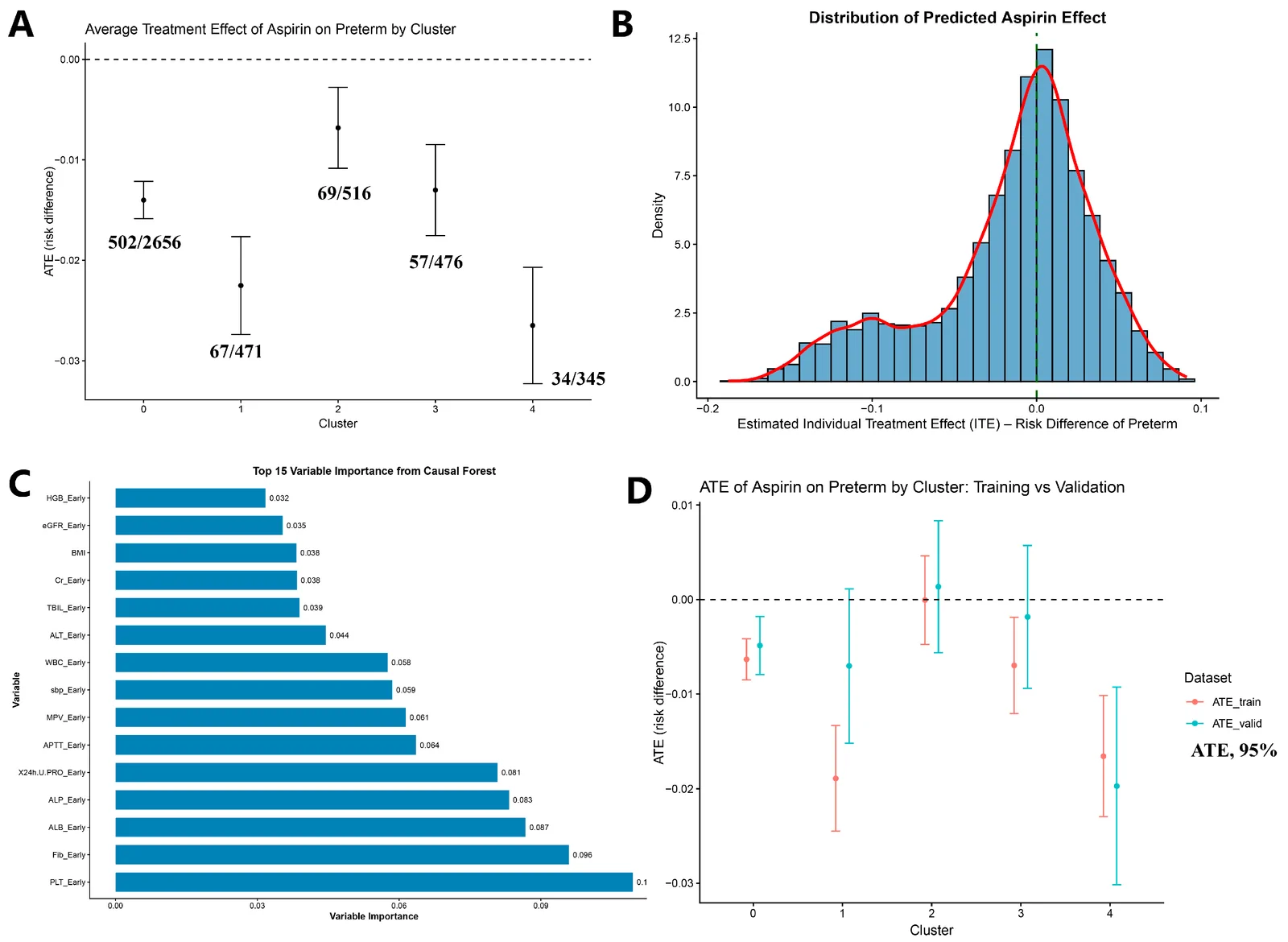
Causal forest results of aspirin for preterm birth < 37 weeks prevention by cluster after clustering using 15 indicators. (**A**) The ATE for each cluster (the number before the slash indicates the number of aspirin users, and the number after the slash indicates the total number of patients in the cluster). (**B**) The distribution of ITE. (**C**) The top 15 variable importance rankings. (**D**) The ATE and 95% in the training (70%) and validation (30%) sets.

After adjusting for maternal age, pre-pregnancy BMI, parity, gravidity, and the 15 early-pregnancy indicators, the ATE for Cluster 4 was −0.026 (95% CI: −0.032 to −0.021), as shown in Figure 3A. The individual effects of aspirin on preterm birth (<37 weeks) in PE patients are presented in Figure 3B, which displays a bimodal distribution, suggesting that a subset of PE patients derive greater benefit from aspirin. The importance ranking of early-pregnancy variables from the causal forest is shown in Figure 3C, with early-pregnancy platelet count ranking highest. After randomly splitting all PE patients into training (70%) and validation (30%) sets, the causal forest model was trained on the training set and then applied to predict the preventive effect of aspirin on preterm birth in the validation set; Cluster 4 again showed the largest estimated reduction, as shown in Figure 3D.

After changing the outcome to preterm birth <34 weeks, the preventive effect of aspirin was weaker than that for preterm birth < 37 weeks, with Cluster 4 still showing the best response (ATE = −0.016, 95% CI: −0.022 to −0.010), and the effect was consistent across the training and validation sets (Supplemental Figure S2). After retraining and clustering using 34 indicators and adjusting for maternal age, pre-pregnancy BMI, parity, gravidity, and the 34 early-pregnancy indicators, causal forest again estimated the largest treatment effect in the subgroup characterized by liver enzyme elevation and coagulation abnormalities (Cluster 4) had the best response to aspirin for preterm birth prevention in PE patients, with consistent results in the training and validation sets (Supplemental Figure S4). Subsequently, in non-PE pregnant women, LSTM training using the 15 indicators followed by causal forest showed no preventive effect of aspirin on preterm birth, with all ATEs close to 0 (ranging from 0.003 to 0.001), as shown in Supplemental Figure S5. In summary, causal forest estimated a larger aspirin-associated reduction in preterm birth in PE patients characterized by liver enzyme elevation and coagulation abnormalities.

Clinical characteristics for each cluster are shown in Table 2. Among them, Cluster 4 had the highest proportion of preterm birth (57.7%) and the highest rate of ICU admission (9.6%). In addition, Cluster 2 had the lowest proportion of preterm birth (26.7%) and the lowest ICU admission rate (1.4%). Causal forest showed that aspirin was associated with the smallest estimated reduction in preterm birth in Cluster 2 (ATE = −0.007, 95% CI: −0.011 to −0.003). Due to the cost of testing, the proportions of placental growth factor (PLGF) and soluble fms-like tyrosine kinase-1 (sFlt-1) measurements were very low across all clusters, as shown in Table 2.

Within-cluster covariate balance and overlap were assessed. SMD values are shown in Supplementary Table S2, and propensity score distributions are shown in Supplementary Table S3 and Figure S7. Overlap was adequate in most clusters, with common support regions ranging from 0.056–0.697 (Cluster 0), 0.050–0.410 (Cluster 1), 0.048–0.609 (Cluster 2), 0.029–0.490 (Cluster 3), and 0.036–0.354 (Cluster 4). However, Cluster 4 included only 37 aspirin-exposed patients, and its overlap was narrower, so the ATE estimate for this cluster should be interpreted with caution.

## 4. Discussion

In this large retrospective cohort study integrating longitudinal pregnancy data with LSTM and causal forest, we identified five potential PE subtypes. One subtype (Cluster 4), characterized by mild liver enzyme elevation and coagulation abnormalities, showed a significant reduction in preterm birth (<37 weeks) with aspirin use (ATE = −0.026, 95% CI: −0.032 to −0.021). This finding was robust across multiple sensitivity analyses: 100-bootstrap resampling yielded a Jaccard similarity of 0.998 for each cluster; 10 repeated LSTM trainings produced a mean ARI of 0.903; using 34 indicators (missing rate < 50%) independently reproduced a similar subtype; and no such benefit was observed in non-preeclamptic women. Moreover, although we attempted multiple imputation to handle missing values, it led to unstable clustering (mean ARI = 0.18 after 10 bootstrap iterations), supporting the validity of our primary −999 masking approach [18]. These results indicate that the heterogeneity of PE extends to aspirin responsiveness, and that routine laboratory markers can identify patients most likely to benefit.

Current classification of PE mainly relies on the timing of onset (early vs. late) or delivery (preterm vs. term). However, the pattern of organ involvement varies substantially among individuals. Several studies have attempted to subtype PE using cross-sectional laboratory data, identifying clusters such as renal impairment, liver dysfunction, coagulopathy, or cardiac injury [4]. Other studies have used latent growth models with a single indicator, such as blood pressure or placental growth factor, to classify PE into different risk subtypes [5,7,25]; however, these models rely on few indicators and are highly sensitive to missing data. To our knowledge, ours is the first study to use longitudinal (two-time windows) multi-indicator data combined with LSTM-based deep learning and causal forest to identify aspirin-responsive subtypes. Alternative clustering methods, including delta features, trajectory clustering, latent class models, and hierarchical clustering, were not formally compared with the LSTM approach in the current study because only two-time windows were available. This limits the methodological novelty of our study. Formal comparisons with these simpler and more interpretable methods should be a requirement for future validation studies.

The absolute risk reduction of 2.6% (ATE = −0.026) corresponds to a NNT of approximately 38. In Cluster 4, with a baseline preterm birth rate of 57.7%, this means one preterm birth prevented per 38 treated women. In contrast, Cluster 2 (with the lowest preterm rate, 26.7%) showed only a minimal response (ATE = −0.007, NNT = 167). In Rolnik et al. [16], combining ASPRE and SPREE (51,024 pregnancies), the NNT for preventing preterm preeclampsia depended on first-trimester risk and adherence: at a risk of 1/50–1/100, NNT was 73–146 with high adherence and 161–321 with 50% adherence; at lower risk, NNT exceeded several thousand. Our NNT of 38 is comparable or more favorable, although our analysis was restricted to women who already developed preeclampsia. However, this estimate should be interpreted cautiously because the analysis was observational, Cluster 4 was relatively small, and the number of aspirin-exposed patients was limited. The narrow confidence interval may not fully reflect uncertainty due to unmeasured confounding. Therefore, this finding is hypothesis-generating and requires independent prospective validation.

The mechanisms by which aspirin prevents PE are likely multifaceted. Current studies indicate that, in addition to its antiplatelet effects [26,27], aspirin can also act directly on trophoblasts [28,29,30,31]. Activation of platelets and the coagulation system have been well documented in PE [32]. Beyond their role in coagulation, platelets are increasingly recognized for their immune and inflammatory functions [33]. In PE, activated platelets not only contribute to placental micro-thrombosis but also interact with trophoblasts [34] and immune cells [35], thereby participating in the pathogenesis of the disease. As a classic antiplatelet agent, aspirin’s ability to prevent PE through platelet inhibition cannot be overlooked. Indeed, in our study, early-pregnancy platelet counts also emerged as the strongest predictor of treatment effect heterogeneity.

Early-pregnancy platelet count ranking highest in our causal model is consistent with the central role of platelet activation in the pathogenesis of preeclampsia. Platelets contribute not only to hemostasis but also to thrombo-inflammation by releasing mediators such as P-selectin, platelet factor 4, thromboxane A2, and soluble CD40 ligand, which can promote endothelial dysfunction, trophoblast injury, and placental micro-thrombosis [36]. Given current knowledge of platelet function, two explanations for the heterogeneity of aspirin’s preventive effect are possible: (1) aspirin reduces platelet activation but does not diminish the immune and inflammatory functions of platelets; (2) some patients do not present with platelet activation as the dominant feature but rather exhibit an inflammatory phenotype. Overall, the subgroup that showed a favourable response to aspirin may be characterised by activation of platelets and the coagulation system, accompanied by hepatic microvascular involvement. Future prospective studies could include more markers of platelet and coagulation activation, placental development indicators, additional time points, and reduced missing data to identify the subtype characterized by coagulation activation and rising liver enzymes, thereby determining the optimal timing for aspirin initiation at earlier gestational weeks and guiding its use in clinical practice for patients with PE.

This study has limitations of being a single-center retrospective study. A stronger design would identify clusters using only the training set. First, as a single-center retrospective study, these findings require validation in independent cohorts to establish their external validity and generalizability [37]. Second, although we used causal forest, unmeasured confounding (e.g., aspirin dosage, adherence, or over-the-counter use) cannot be entirely excluded. In this retrospective cohort, detailed information on aspirin exposure was not reliably available, including dosage, gestational age at initiation, duration of treatment, adherence, over-the-counter use, and the clinical indication for prescription. These missing data may lead to exposure misclassification and residual confounding, because women who were prescribed aspirin may differ systematically from those who were not in ways not fully captured by the covariates included in the causal forest. Although we adjusted for maternal age, pre-pregnancy BMI, parity, gravidity, diabetes, hypertension, and early-pregnancy indicators, we cannot rule out bias from unmeasured differences in aspirin use patterns or clinician prescribing practice [20,37,38]. Therefore, the estimated aspirin effects should be interpreted cautiously and regarded as hypothesis-generating. We also assessed covariate balance and propensity score overlap within each cluster. Although overlap was adequate in most clusters, Cluster 4 had limited overlap because only 37 patients were aspirin-exposed. This means the estimated ATE for Cluster 4 remains vulnerable to instability, residual confounding, and limited positivity [22]. Future prospective studies should standardize the recording of aspirin dose, timing of initiation, duration, adherence, and indication to enable more reliable causal inference. Third, we had only two time points per patient. This is important because aspirin prophylaxis is typically initiated before 16 weeks of gestation (12–28 weeks) [39,40]. Therefore, a subtype defined using measurements obtained within 2 weeks before delivery cannot be directly used to guide early aspirin initiation. This late-pregnancy phenotype may reflect disease progression, treatment effects, or both, as most patients did not meet HELLP diagnostic criteria and only approximately 10% were diagnosed with HELLP syndrome (34/345). Other studies that used only early-pregnancy indicators to build clinical risk scores have not performed well in clinical practice [41]. In contrast, based on our findings, longitudinal trajectory models of indicators that ranked highly in the causal forest (such as fibrinogen and platelet count) may be more informative than single-time-point measurements. Future studies with multiple early-pregnancy time points are needed to determine whether early platelet/coagulation trajectories can identify aspirin-responsive women before prophylaxis is initiated. Such studies could be designed within a target trial emulation framework, in which eligibility, treatment assignment time, aspirin initiation window, follow-up start, outcome window, confounder assessment, and analysis plan are explicitly defined. This would help clarify whether early platelet/coagulation trajectories can guide the timing of aspirin initiation [42,43]. Fourth, PLGF and sFlt-1 were not included in the clustering indicators, and the proportion of patients with measurements was very low across all clusters. In routine obstetric practice at our center, these markers are not universally measured because of cost and low clinical cost-effectiveness. Therefore, we could not evaluate the role of angiogenic pathways in this study. Finally, although the −999 masking strategy has potential bias, it was more suitable for this dataset than imputation. Masked MSE excluded missing positions from the loss, and multiple imputation yielded unstable clustering. However, this instability should be interpreted as a warning that clustering is sensitive to missing-data handling, not as evidence that the −999 strategy is valid. The −999 placeholder still entered the LSTM and could affect the latent representation, so this strategy may not suit all missingness patterns. Missing-data handling is dataset-dependent and should be compared across methods. These findings suggest that the cluster structure may partly depend on the missing-data strategy. These findings require further validation in independent, multicenter, prospective cohorts, with standardized recording of aspirin exposure and comparison of alternative missing-data approaches before any clinical application.

## 5. Conclusions

In summary, we identified a PE subtype characterized by persistent mild liver enzyme elevation and coagulation abnormalities (a phenotype that requires further validation in studies with multiple time points). This subtype showed a larger estimated reduction in preterm birth associated with aspirin prophylaxis. This finding was robust across multiple sensitivity analyses but should be interpreted as hypothesis-generating. Notably, sustained liver enzyme elevation or coagulation activation may be associated with greater aspirin benefit; however, this requires validation in independent prospective cohorts before it can inform personalized prevention strategies for PE.

## Figures and Tables

**Table 1:** Population baseline characteristics.

Variable	PE *n* = 4505	Non-PE *n* = 48,857	
Age, years	33 (31–36)	33 (30–36)	*p* < 0.001
Body mass index, BMI, kg/m^2^	23.9 (21.5–26.7)	21.7 (20.0–23.9)	*p* < 0.001
Gestational days	260 (244–270)	274 (267–280)	*p* < 0.001
Placental abruption	136 (3%)	310 (0.6%)	*p* < 0.001
Diabetes	1630 (36.2%)	13,374 (27.4%)	*p* < 0.001
Hypertension	1160 (25.7%)	3929 (8%)	*p* < 0.001
ICU admission rate	140 (3.1%)	273 (0.6%)	*p* < 0.001
Multiple pregnancy	1135 (25.2%)	4045 (8.3%)	*p* < 0.001
Stillbirth	108 (2.4%)	676 (1.4%)	*p* < 0.001
Aspirin use rate	732 (16.25%)	4711 (9.64%)	*p* < 0.001
Blood loss, mL	730 (570–1060)	650 (550–890)	*p* < 0.001
Red blood cell transfusion, Units	2 (2–4)	2 (2–4)	*p* = 0.401
Plasma transfusion, mL	400 (400–800)	400 (400–800)	*p* = 0.493
Platelet transfusion, Units	1 (1–2)	1 (1–2)	*p* = 0.233

**Table 2:** ATE results and clinical characteristics of each cluster.

	Cluster 0 *n* = 2696	Cluster 1 *n* = 471	Cluster 2 *n* = 516	Cluster 3 *n* = 476	Cluster 4 *n* = 345
Diabetes	1004 (37.2%)	166 (35.2%)	208 (40.3%)	149 (31.3%)	103 (29.9%)
Hypertension	753 (27.9%)	111 (23.6%)	109 (21.1%)	98 (20.6%)	89 (25.8%)
Body Mass Index, Kg/m^2^ (Median, quartile)	23.9 (21.45, 26.8)	24.2 (22.0, 27.2)	23.8 (21.5, 26.7)	23.2 (20.9, 26.0)	23.9 (21.4, 27.1)
Age, years (Median, quartile)	33 (31, 36)	33 (30, 36)	34 (31, 36)	33 (30, 37)	34 (31, 37)
Gravidity (Median, quartile)	1 (1, 2)	2 (1, 3)	2 (1, 3)	1 (1, 2)	2 (1, 3)
ATE, 95% CI	−0.014 (−0.016, −0.012)	−0.023 (−0.027, −0.018)	−0.007 (−0.011, −0.003)	−0.013 (−0.018, −0.008)	−0.026 (−0.032, −0.021)
ICU admission rate %	2.4	3.0	1.4	4.6	9.6
preterm birth < 37 weeks %	52.1	45.4	27.7	44.7	57.7
preterm birth < 34 weeks %	22.0	20.2	7.2	16.6	26.7
severe preeclampsia %	31.6	42.7	22.3	36.1	37.7
PLGF, pg/mL	*n* = 72; 269,696.6 (49.7–197.8)	*n* = 3; 168 (93.7–838.5)	*n* = 6; 77.4 (38.1–128.5)	*n* = 11; 47,661.4 (51.2–79.6)	*n* = 6; 122.1 (48.8–239.2)
sFlt-1, pg/mL	*n* = 4; 3430 (2205.2–4426.8)	*n* = 0	*n* = 1; 13,179	*n* = 0	*n* = 1; 2332
Aspirin use	502 (18.6%)	67 (14.2%)	69 (13.3%)	57 (12.0%)	37 (10.7%)
HELLP syndrome	57 (2.1%)	16 (3.4%)	5 (1.0%)	25 (5.3%)	34 (9.9%)

## Data Availability

The datasets generated and/or analysed during the current study are available from the corresponding author on reasonable request.

## Supplementary Materials

The following supporting information can be downloaded at: https://www.mdpi.com/article/10.3390/bioengineering13101171/s1. Table S1. Missing rates of indicators used in the study. Supplemental Table S2. Standardized mean differences for baseline covariates between aspirin-exposed and unexposed women within each preeclampsia subtype. Supplemental Table S3. Propensity score distribution by preeclampsia subtype and aspirin exposure. Supplemental Figure S1. Changes in 15 indicators across the two time windows by cluster. Supplemental Figure S2. Causal forest results of aspirin for preterm birth < 34 weeks prevention by cluster after clustering using 15 indicators. Supplemental Figure S3. LSTM training and clustering results using 34 indicators. Supplemental Figure S4. Causal forest results of aspirin for preterm birth < 37 weeks prevention by cluster after clustering using 34 indicators. Supplemental Figure S5. LSTM training and causal forest results in non-PE pregnant women using 15 indicators. Supplemental Figure S6. LSTM training and clustering results based on 15 indicators after multiple imputation. Supplementary Figure S7. Propensity score distributions for aspirin-exposed and unexposed women within each preeclampsia subtype.
