# Supplementary material for: Using Long Short-Term Memory and Causal Forest to Identify Preeclampsia Subtypes with Differential Aspirin Associations for Preterm Birth Prevention
Source: Bioengineering (Basel). 2026 Oct 8;13(10):1171. doi: 10.3390/bioengineering13101171 (PMC13701992; doi:10.3390/bioengineering13101171)
Supplement: Supplementary file 1 [file bioengineering-13-01171-s001.zip › Supplementary Material.pdf]

Supplemental Table S1. Missing rates of indicators used in the study.

Supplemental Table S2. Standardized mean differences for baseline covariates between aspirin-exposed and unexposed women within each preeclampsia subtype.

Supplemental Table S3. Propensity score distribution by preeclampsia subtype and aspirin exposure.

Supplemental Figure S1. Changes in 15 indicators across the two-time windows by cluster.

Supplemental Figure S2. Causal forest results of aspirin for preterm birth <34 weeks prevention by cluster after clustering using 15 indicators.

Supplemental Figure S3. LSTM training and clustering results using 34 indicators.

Supplemental Figure S4. Causal forest results of aspirin for preterm birth <37 weeks prevention by cluster after clustering using 34 indicators.

Supplemental Figure S5. LSTM training and causal forest results in non-PE pregnant women using 15 indicators.

Supplemental Figure S6. LSTM training and clustering results based on 15 indicators after multiple imputation.

Supplementary Figure S7. Propensity score distributions for aspirin-exposed and unexposed women within each preeclampsia subtype.

Supplemental Table S1. Missing rates of indicators used in the study.

Category	Features	Number of missing instances (%) in PE patients, n = 4505	Number of missing instances (%) in non-PE pregnant women, n = 48857
Demographics	Age, Gravidity, Parity, Gestational days	0	0
	Pre-pregnancy BMI	6.66	9.37
Laboratory indicators	24h.U.PRO < 16 weeks	92.85	96.33
	sbp < 16 weeks	66.74	71.65
	PLCR < 16 weeks	35.32	
	PCT < 16 weeks	35.3	
	MPV < 16 weeks	35.3	20.83
	PDW < 16 weeks	35.3	
	RDW SD < 16 weeks	35.15	
	RDW CV < 16 weeks	35.12	
	WBC < 16 weeks	35.1	20.55
	HGB < 16 weeks	35.1	20.55
	MCV < 16 weeks	35.1	
	MCH < 16 weeks	35.1	
	MCHC < 16 weeks	35.1	
	LYMPH < 16 weeks	35.1	
	Neut < 16 weeks	35.1	
	EO < 16 weeks	35.1	
	BASO < 16 weeks	35.1	
	MONO < 16 weeks	35.1	
	ALP < 16 weeks	26.82	60.93
	Ca < 16 weeks	26.49	
	GGT < 16 weeks	26.33	
	TP < 16 weeks	26.31	
	ALB < 16 weeks	26.27	11.8
	UA < 16 weeks	26	
	eGFR < 16 weeks	25.98	11.68
	PT < 16 weeks	25.78	
	Fib < 16 weeks	25.78	11.24
	APTT < 16 weeks	25.78	11.23
	TT < 16 weeks	25.78	
	Cr < 16 weeks	25.78	11.51
	ALT < 16 weeks	25.58	11.36
	TBIL < 16 weeks	25.56	11.35
	AST < 16 weeks	25.56	

	PLT < 16 weeks	24.87	10.94
	D-dimer < 16 weeks	78.66	76.74
	GGT 2 weeks before delivery	23.09	
	24h.U.PRO 2 weeks before delivery	22.31	86.46
	eGFR 2 weeks before delivery	16.94	65.65
	UA 2 weeks before delivery	11.99	
	sbp 2 weeks before delivery	8.61	26.96
	TP 2 weeks before delivery	5.99	
	ALT 2 weeks before delivery	5.22	60.95
	ALB 2 weeks before delivery	5.13	61.15
	Ca 2 weeks before delivery	5.13	
	ALP 2 weeks before delivery	5	60.93
	TBIL 2 weeks before delivery	4.97	60.15
	Cr 2 weeks before delivery	4.66	61.24
	AST 2 weeks before delivery	4.53	
	PCT 2 weeks before delivery	1.58	
	MPV 2 weeks before delivery	1.58	1.73
	PDW 2 weeks before delivery	1.58	
	PLCR 2 weeks before delivery	1.58	
	RDW CV 2 weeks before delivery	0.64	
	RDW SD 2 weeks before delivery	0.64	
	WBC 2 weeks before delivery	0.62	1.21
	HGB 2 weeks before delivery	0.62	1.21
	MCV 2 weeks before delivery	0.62	
	MCH 2 weeks before delivery	0.62	
	MCHC 2 weeks before delivery	0.62	
	LYMPH 2 weeks before delivery	0.62	
	Neut 2 weeks before delivery	0.62	
	EO 2 weeks before delivery	0.62	
	BASO. 2 weeks before delivery	0.62	
	MONO. 2 weeks before delivery	0.62	
	TT 2 weeks before delivery	0.36	
	PT 2 weeks before delivery	0.31	
	Fib 2 weeks before delivery	0.29	0.86
	APTT 2 weeks before delivery	0.29	0.86
	PLT 2 weeks before delivery	0.11	0.49
	D-dimer 2 weeks before delivery	9.81	43.80

Supplemental Table S2. Standardized mean differences for baseline covariates between aspirin-exposed and unexposed women within each preeclampsia subtype.

cluster	variable	Unadjusted Difference
0	age	0.276477
0	BMI	0.131541
0	BMI:<NA>	0.001554
0	g	0.19804
0	p	-0.18739
0	DM	0.095596
0	HP	0.175729
0	PEH	-0.00046
1	age	0.222184
1	BMI	0.019176
1	BMI:<NA>	0.062842
1	g	0.049067
1	p	-0.0968
1	DM	0.076326
1	HP	0.247266
1	PEH	0
2	age	0.48105
2	BMI	-0.00442
2	BMI:<NA>	0.024219
2	g	0.441904
2	p	0.152522
2	DM	0.036572
2	HP	0.207859
2	PEH	-0.00224
3	age	0.342421
3	BMI	0.005924
3	BMI:<NA>	-0.0031
3	g	0.367887
3	p	-0.13007
3	DM	0.023071
3	HP	0.124859
3	PEH	0
4	age	0.473146
4	BMI	0.128927
4	BMI:<NA>	-0.03686
4	g	0.390918
4	p	0.13767
4	DM	0.180239
4	HP	0.225693
4	PEH	-0.00325

Supplemental Table S3. Propensity score distribution by preeclampsia subtype and aspirin exposure.

Cluster	Aspirin	Mean (SD)	Median (IQR)	Range	Common support
**0**	**No**	0.175 (0.09)	0.149 (0.114–0.215)	0–0.697	0.056–0.697
**0**	**Yes**	0.233 (0.109)	0.208 (0.152–0.289)	0.056–0.838	
**1**	**No**	0.125 (0.080)	0.097 (0.079–0.134)	0.027–0.410	0.050–0.410
**1**	**Yes**	0.185 (0.100)	0.139 (0.101–0.277)	0.050–0.448	
**2**	**No**	0.118 (0.086)	0.088 (0.065–0.140)	0–0.609	0.048–0.609
**2**	**Yes**	0.213 (0.173)	0.177 (0.095–0.244)	0.048–0.895	
**3**	**No**	0.113 (0.069)	0.092 (0.070–0.135)	0.021–0.490	0.029–0.490
**3**	**Yes**	0.171 (0.109)	0.146 (0.093–0.214)	0.029–0.513	
**4**	**No**	0.104 (0.069)	0.080 (0.049–0.140)	0–0.354	0.036–0.354
**4**	**Yes**	0.169 (0.116)	0.148 (0.093–0.207)	0.036–0.574	

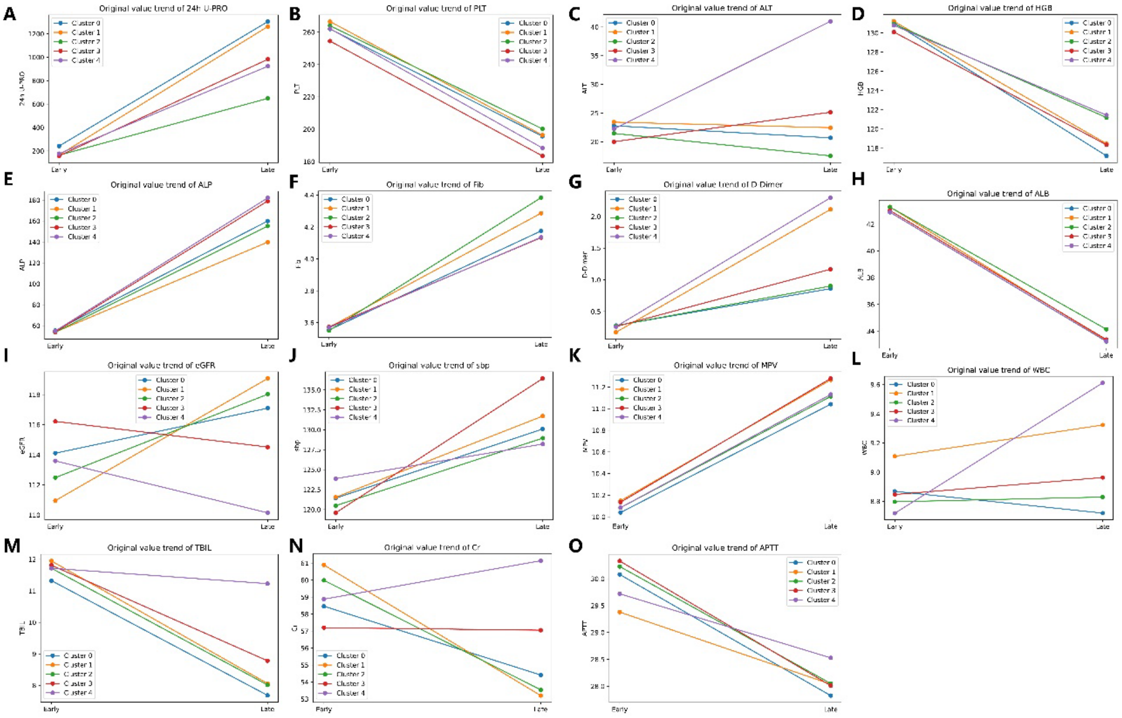

Supplemental Figure S1. Changes in 15 indicators across the two time windows by cluster.

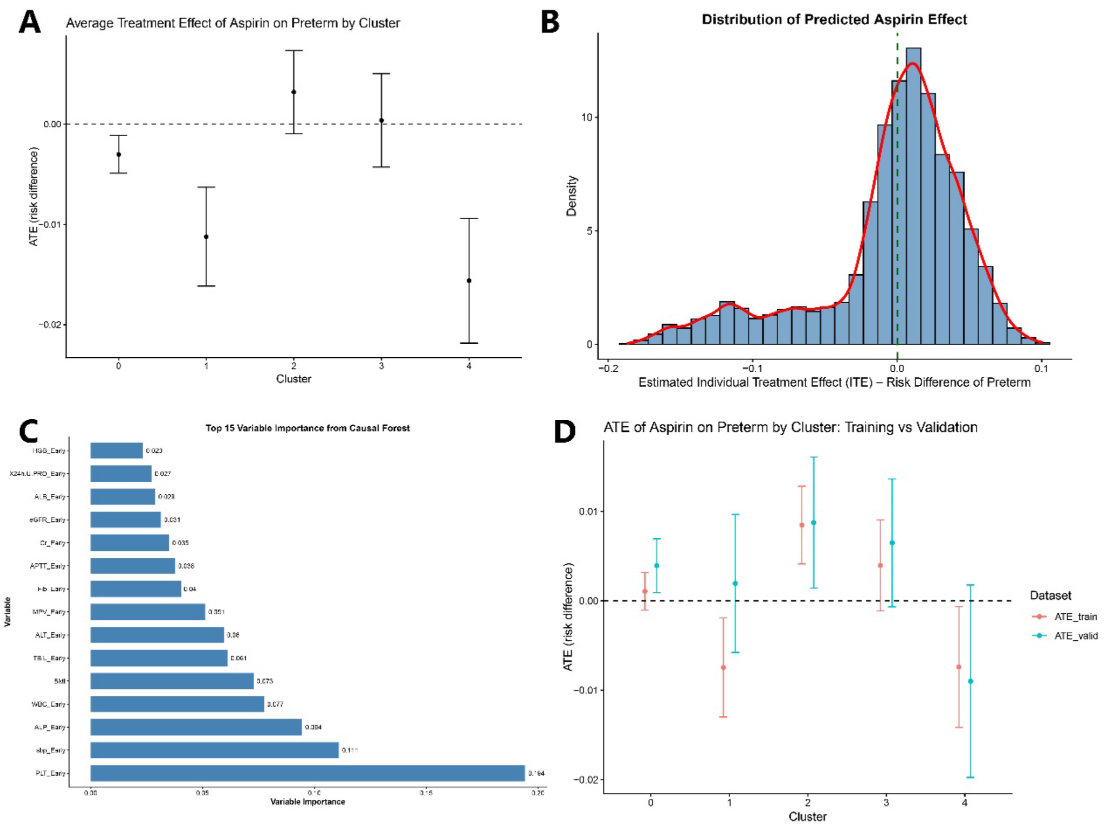

Supplemental Figure S2. Causal forest results of aspirin for preterm birth <34 weeks prevention by cluster after clustering using 15 indicators. A, the average treatment effect (ATE) for each cluster; B, the distribution of individual treatment effects (ITE); C, the top 15 variable importance rankings; D, the ATE in the training (70%) and validation (30%) sets.

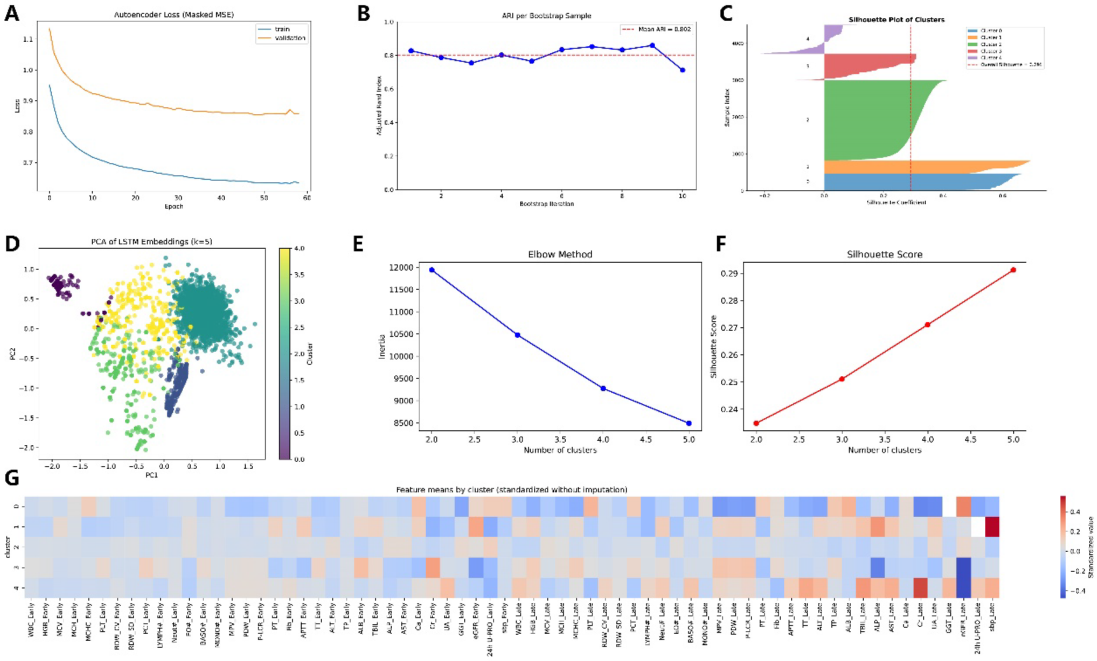

Supplemental Figure S3. LSTM training and clustering results using 34 indicators. A, training mean squared error (MSE); B, adjusted Rand index (ARI) calculated from 10 LSTM retraining repeats; C, silhouette coefficient for each cluster; D, principal component analysis (PCA) visualization of the 34 indicators embedding space; E-F, determination of the optimal number of clusters based on the silhouette coefficient; G, heatmap of the 34 indicators across the five clusters.

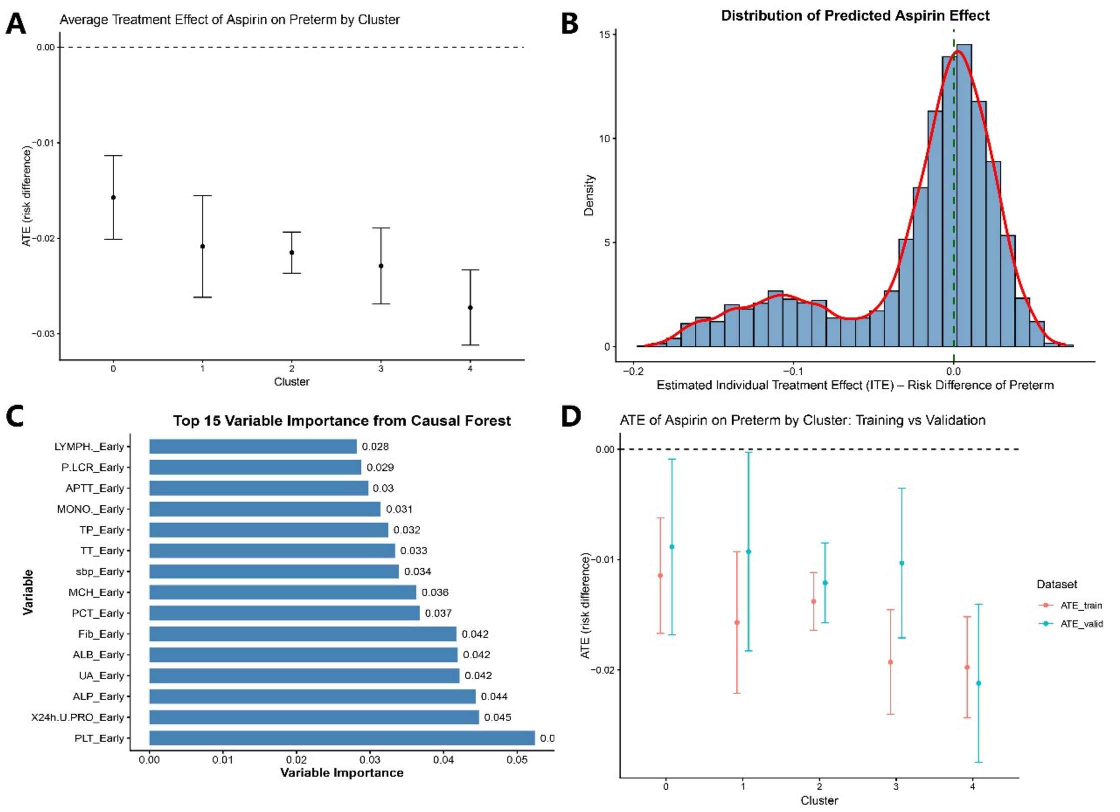

Supplemental Figure S4. Causal forest results of aspirin for preterm birth <37 weeks prevention by cluster after clustering using 34 indicators. A, the ATE for each cluster; B, the distribution of ITE; C, the top 15 variable importance rankings; D, the ATE in the training (70%) and validation (30%) sets.

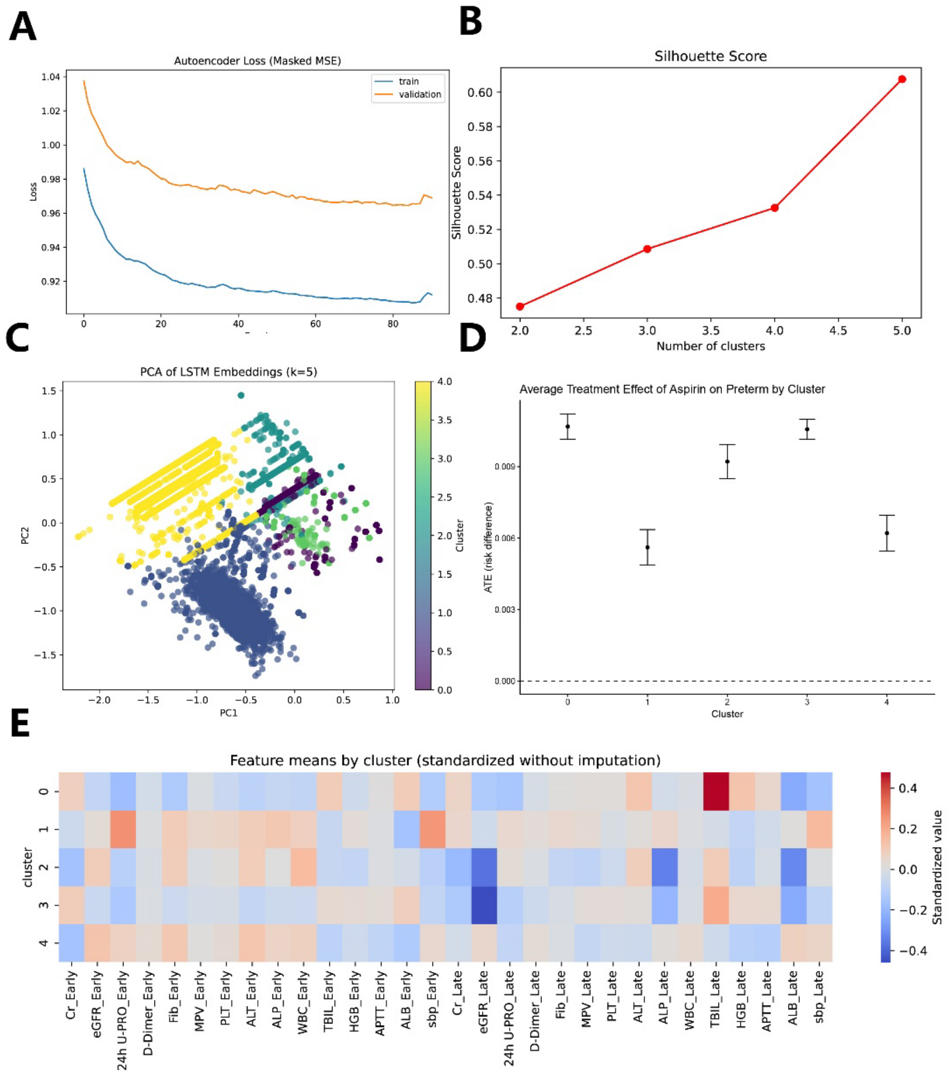

Supplemental Figure S5. LSTM training and causal forest results in non-PE pregnant women using 15 indicators. A, training and validation MSE during LSTM autoencoder training, indicating model convergence without overfitting; B, determination of the optimal number of clusters based on the silhouette coefficient; C, PCA visualization of the 15 indicators embedding space; D, the ATE for each cluster; E, heatmap of the 15 indicators across the five clusters.

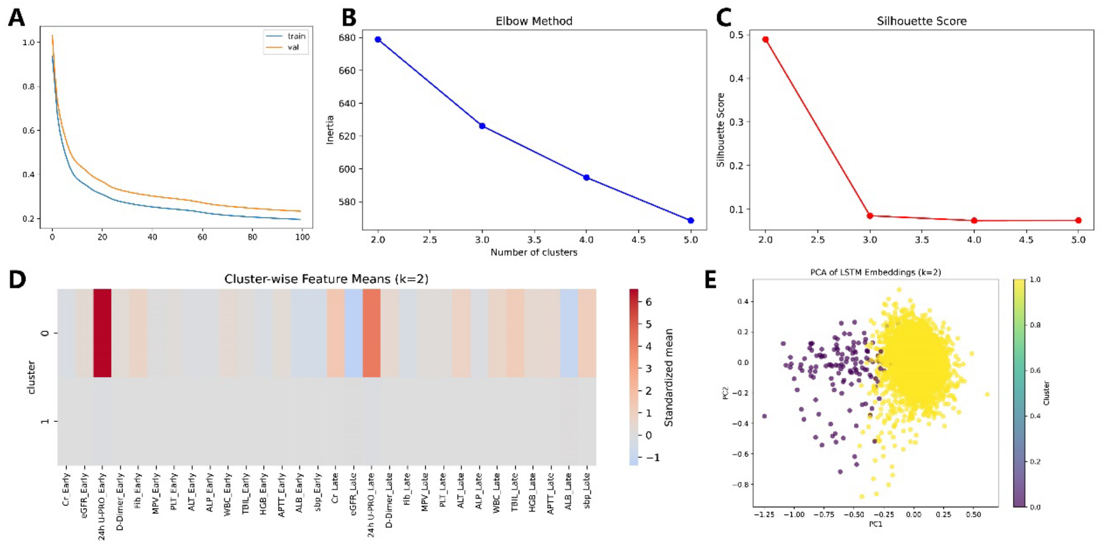

Supplemental Figure S6. LSTM training and clustering results based on 15 indicators after multiple imputation. A, training and validation MSE during LSTM autoencoder training; B-C, determination of the optimal number of clusters based on the silhouette coefficient; D, heatmap of the 15 indicators across the two clusters. E, PCA visualization of the 15 indicators embedding space.

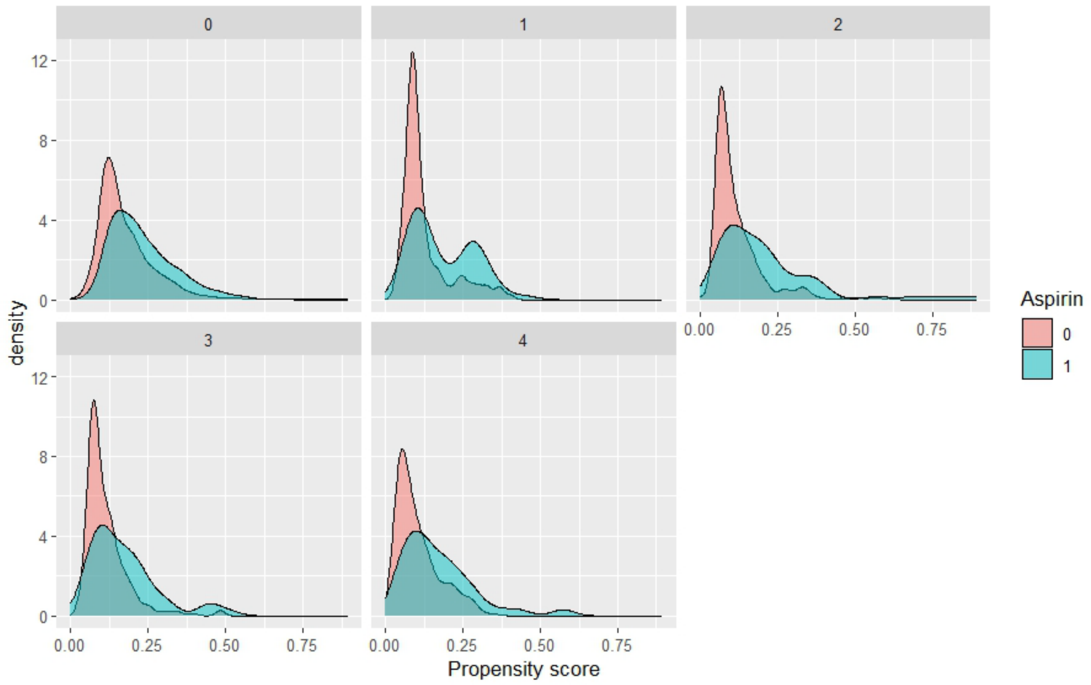

Supplementary Figure S7. Propensity score distributions for aspirin-exposed and unexposed women within each preeclampsia subtype. Each panel represents one cluster (0-4). Red curves indicate women not exposed to aspirin (aspirin = 0); blue curves indicate women exposed to aspirin (aspirin = 1). The x-axis shows the propensity score, estimated using logistic regression with aspirin use as the outcome and age, BMI, gravidity, parity, diabetes, hypertension, and preeclampsia history as covariates.
